# Quantitative evaluation of normal myocardial stress perfusion using Fermi and MMID4

**DOI:** 10.1186/1532-429X-11-S1-P10

**Published:** 2009-01-28

**Authors:** Yi Wang, Paphael Hazel, Bin Luo, Jing Han, Nathaniel Reichek

**Affiliations:** grid.416387.f St. Francis Hospital, Roslyn, NY USA

**Keywords:** Myocardial Stress Perfusion Imaging, Adenosine Infusion, Coronary Calcium Score, Myocardial Stress Perfusion, Stress Perfusion Imaging

## Background

MR first pass perfusion quantification has been demonstrated to be a powerful technique for diagnosing cardiac perfusion deficits, since its introduction in the early 1990s. Model based MR perfusion quantification is feasible and may provide a sensitive absolute perfusion evaluation tool alternative to PET. However, MR perfusion quantification results vary depending on the model used, imaging sequences, and contrast dosage etc.

## Purpose

We compared MMID4 and Fermi model-based quantitative methods for myocardial stress perfusion imaging on 16 normal volunteers. The goals of our study were to define normal quantification results and the relationship between Fermi and MMID4 methods, as well as the impact of age on normal myocardial perfusion.

## Methods

To ensure normality of the volunteers (n = 16, ages: 43.9 ± 15.8 years, 5 males) exclusions included: hypertension, diabetes, smoking, family or personal history of cardiac disease and total coronary calcium score < 20 by either EBCT or MDCT.

Contrast first pass perfusion studies under adenosine stress and at rest were performed on a 1.5 T scanner. After 3 minutes of adenosine infusion, long axis perfusion imaging was obtained with Gadodiamide injection at a dose of 0.05 mmol/kg bodyweight. A saturation recovery SSFP sequence was used with 160 ms per slice. TR/TE/TI = 2.9 ms/1.3 ms/90 ms and voxel size 1.9 × 2.8 × 8 mm^3^.

Using MASS (Medis, Leiden, the Netherlands) software, the myocardial contours were drawn, divided into 6 equal segments, and then propagated through all time point. Mean signal intensities in each myocardial segment at every time point were saved in text files. A custom developed program read the data file and calculated absolute perfusion based on both the Fermi deconvolution algorithm and the MMID4 algorithm.

A total 288 segmental perfusion values were evaluated for differences between subjects and algorithms using mixed effect ANOVA. The coefficient of variation (CV) of each algorithm was calculated as SD/mean × 100%.

## Results

The mean heart rates at rest and under stress were 61.9 ± 5.9 and 86.8 ± 16.4 beats per minute, respectively (p < 0.0001). The effect of adenosine infusion was a 40.3 ± 22.5% increase in mean heart rate and no significant decrease in both systolic and diastolic blood pressure. The mean and SD of perfusion reserve for Fermi was 2.61 ± 1.02, and for MMID4 was 2.77 ± 2.08. There were significant differences between Fermi and MMID4 adjusted by slice and segment (F = 28.00, p < 0.0001). However, MMID4 perfusion reserve showed a much greater variations (CV of 119.25) than Fermi (CV = 38.87) (p < 0.0001).

There was a gradual decline with age of MPR quantified by the Fermi model with a linear regression, MPR = 3.6433 – 0.022 × age, and a coefficient of determination of 0.4258 (p = 0.006). The MMID4 results did not demonstrate a significant age effect.

## Conclusion

Both MMID4 and Fermi algorithms can be used for perfusion quantification and their perfusion reserve results in normals are very similar to that published in PET literature. However, MMID4 results are more variable, obscuring the effect of age, and seem to be more susceptible to artifacts.

